# Supplementary material for: Prospective discovery of small molecule enhancers of an E3 ligase-substrate interaction
Source: Nat Commun. 2019 Mar 29;10:1402. doi: 10.1038/s41467-019-09358-9 (PMC6441019; doi:10.1038/s41467-019-09358-9)
Supplement: Supplementary file 1 — Supplementary Information [file 41467_2019_9358_MOESM1_ESM.pdf]

**Supplementary Information**

# Prospective discovery of small molecule enhancers of an E3 ligase-substrate interaction

Simonetta et. al.,

# Supplementary Figure 1

**A**

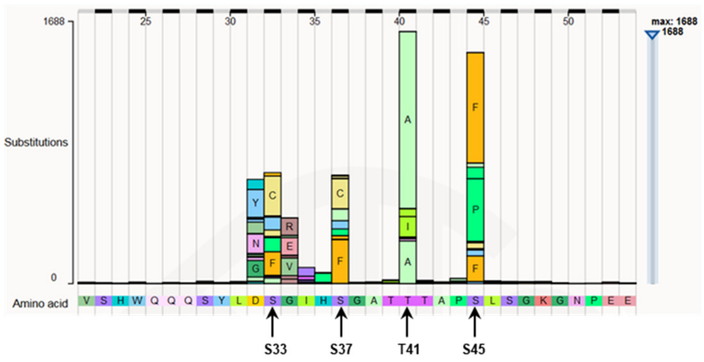


**B**

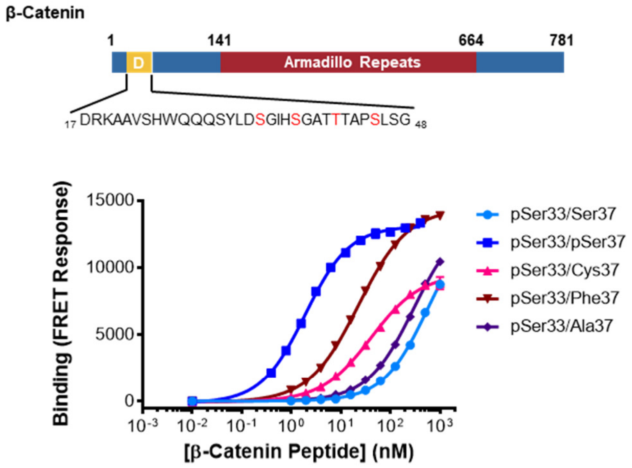


| β-Catenin Peptide | $K_D$ (nM) | Fold binding defect relative to pSer33/pSer37 |
|---|---|---|
| pSer33/pSer37 | 2 | - |
| pSer33/Ser37 | 530 | 265 |
| pSer33/S37C | 154 | 77 |
| pSer33/S37F | 23 | 11.5 |
| pSer33/S37A | 272 | 136 |

**Supplementary Figure 1: β-Catenin phosphodegron mutations impairs its binding to β-TrCP.**

(A) Frequently observed β-Catenin mutations in cancer are shown (data derived from COSMIC).

(B) Binding affinities of various Ser37 β-Catenin mutant peptides were measured in a TR-FRET assay and are reported. Briefly, BODIPY-FL-labeled β-Catenin peptides were titrated against 300 pM β-TrCP complex. Binding affinities were estimated by curve fitting to one-site binding model.

# Supplementary Figure 2

**A**

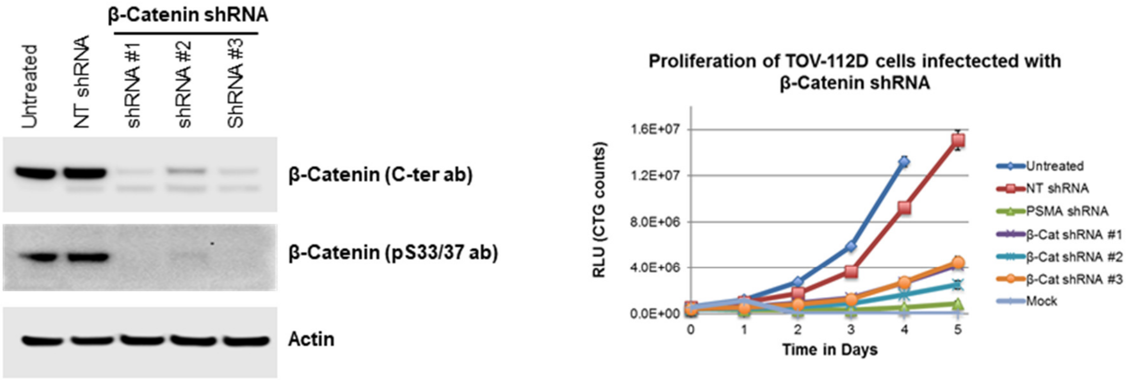


**B**

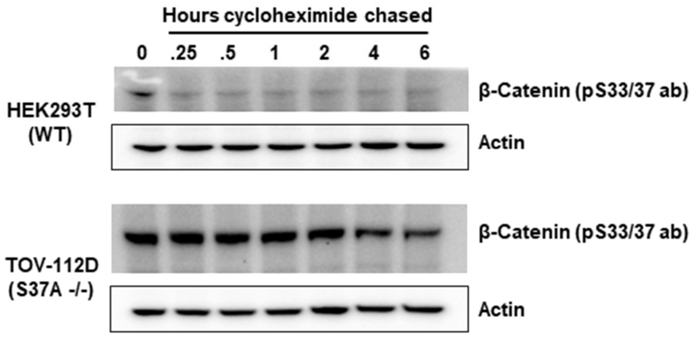


**C**

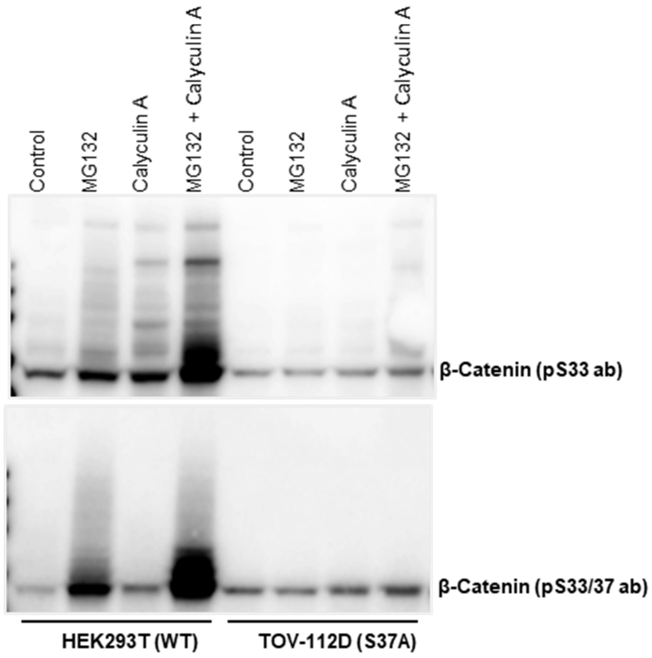

**Supplementary Figure 2: S37A mutant β-Catenin is defective in ubiquitylation and degradation.**

(A) β-Catenin was knocked down in TOV-112D cells and cell proliferation was monitored (right). Knockdown efficiency was evaluated by western analysis of the β-Catenin protein (left).

(B) The half-lives of β-Catenin or S37A mutant β-Catenin were monitored in HEK293T or TOV-112D cells, respectively, following 100 μg/ml cycloheximide treatment. Cells were harvested at time points indicated and analyzed for protein levels be western analysis.

(C) HEK293T or TOV-112D cells were treated with proteasome and/or phosphatase inhibitors (as indicated) and β-Catenin levels were analyzed by western analysis. The high molecular weight smear observed following proteasome inhibitor treatment corresponds to ubiquitylated β-Catenin.

## Supplementary Figure 3

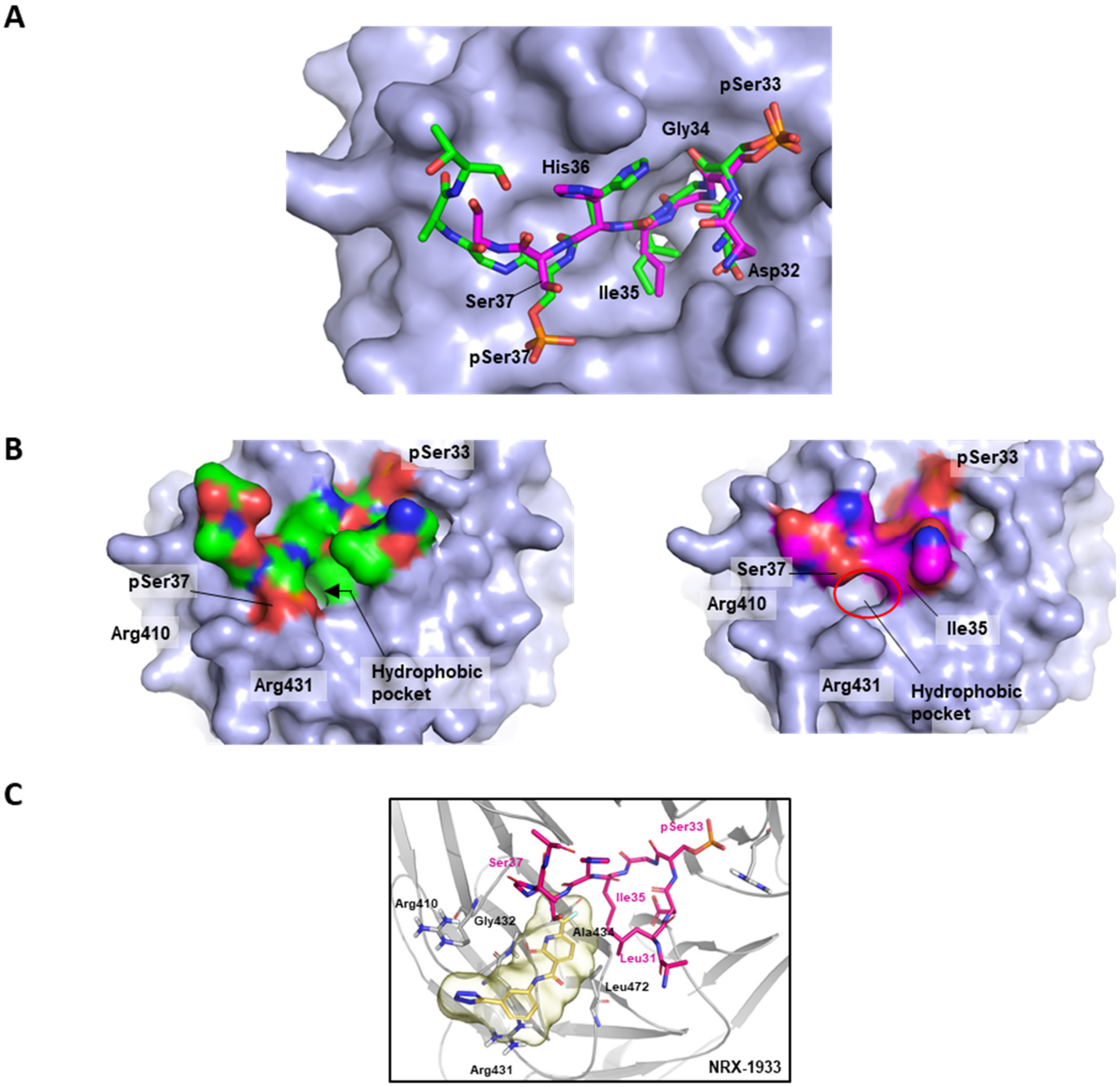


**Supplementary Figure 3: Crystal structure of pSer33/pSer37 and pSer33/Ser37 β-Catenin peptides bound to β-TrCP.**

(A) Overlay of the pSer33/pSer37 β-Catenin peptide (green) (PDB: 1P22)[30] with the monophosphorylated pSer33/Ser37 peptide (magenta) bound to the β-propeller of β-TrCP (light

blue surface). The β-TrCP surface shown is for the pSer33/Ser37 bound structure. The alignment was made by aligning the Cα positions in β-TrCP.

(B) Comparison of the β-TrCP:β-Catenin surface from the pSer33/pSer37 crystal structure (1P22[30], green peptide surface) with that from the pSer33/Ser37 bound structure (magenta peptide surface, light blue β-TrCP surface). Removal of the phosphate group from Ser37 results in exposure of a hydrophobic pocket formed between β-TrCP and β-Catenin (red circle).

(C) Binding of NRX-1933 to the β-Catenin:β-TrCP complex.

# Supplementary Figure 4

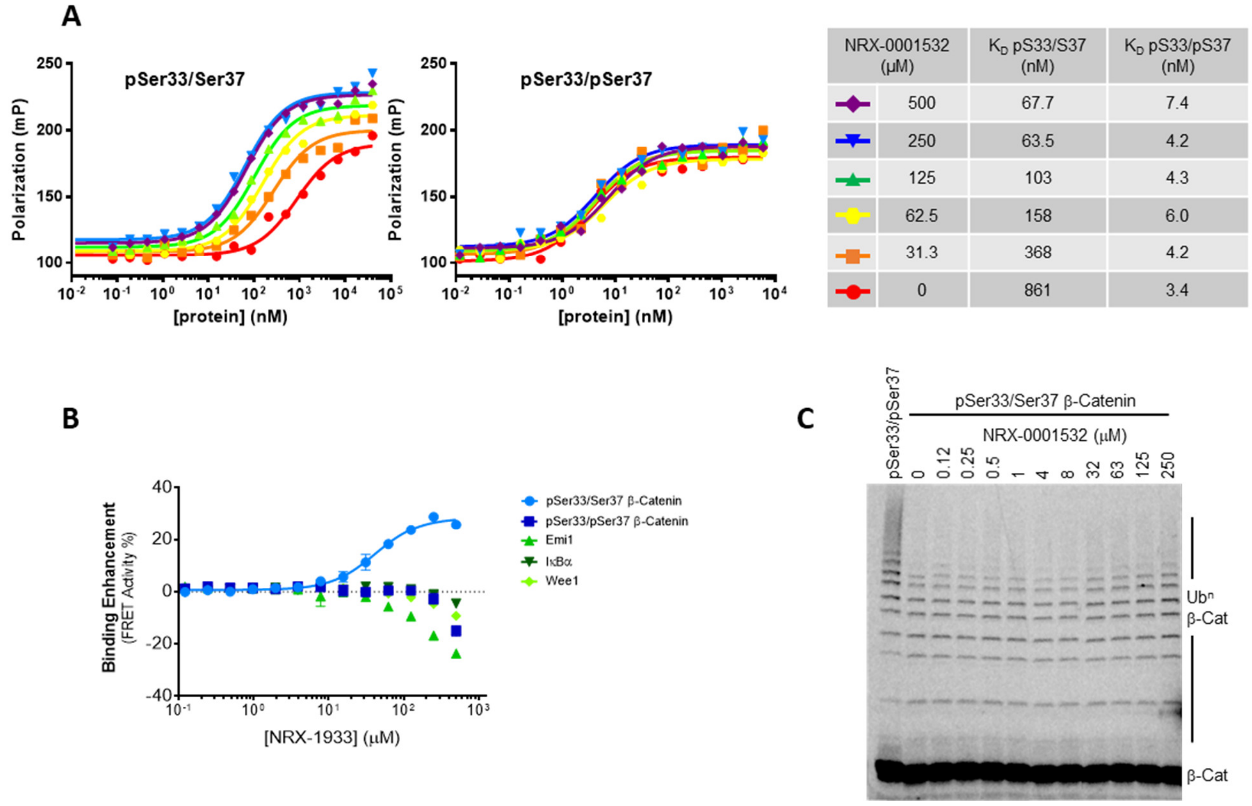


**Supplementary Figure 4: Binding enhancement and ubiquitylation activity of NRX-1532.**

(A) The binding affinities of the pSer33/Ser37 and pSer33/pSer37 β-Catenin peptides to β-TrCP were measured in the FP assay by titrating β-TrCP complex against 5 nM BODIPY-TMR-labeled β-Catenin peptide (residues 17-48) at varying concentrations of NRX-1532. The $K_D$s are reported for both pSer33/Ser37 and pSer33/pSer37 β-Catenin peptides

(B) Enhancement of various β-TrCP substrate peptides binding to β-TrCP with increasing concentrations of NRX-1933 using the TR-FRET binding assay.

(C) Ubiquitylation of fluorescently-labeled β-Catenin peptide (residue 17-60) by SCF$^{\beta\text{-TrCP}}$ with increasing concentrations of NRX-1532.

## Supplementary Figure 5

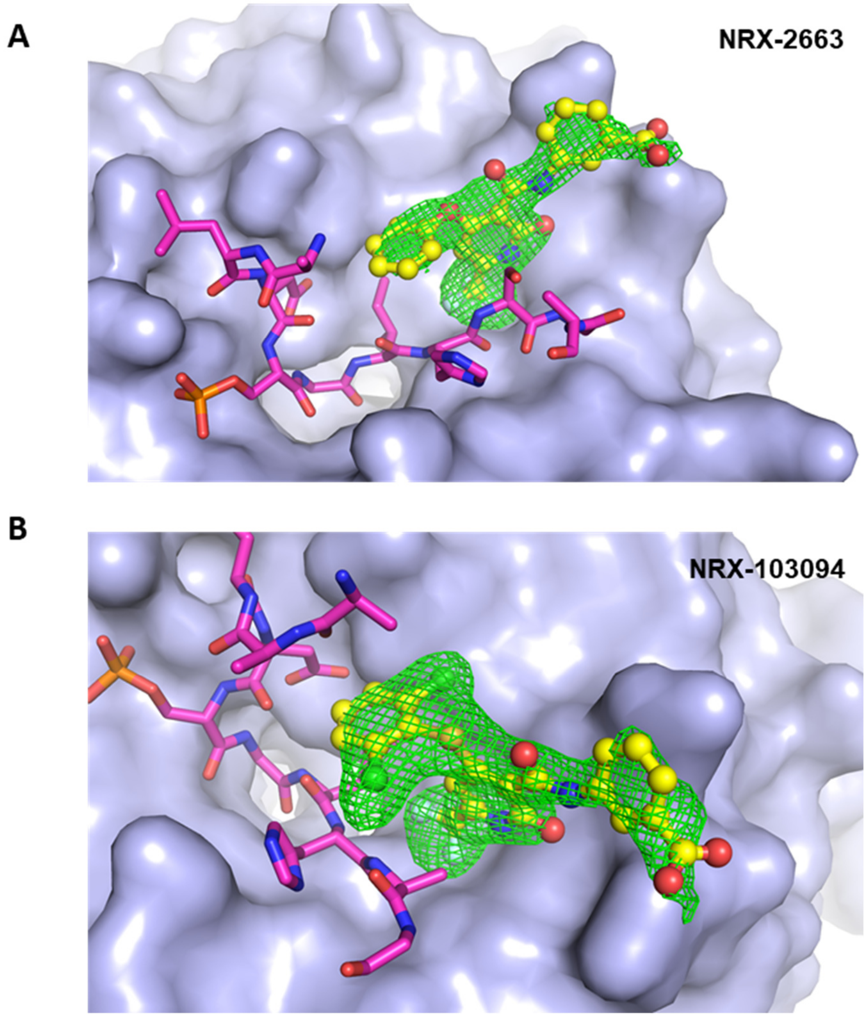


**Supplementary Figure 5: Electron density for NRX-2663 and NRX-103094 bound to the β-Catenin:β-TrCP complex.**

(A) Unbiased Fo-Fc electron density for NRX-2663 at 3.0 standard deviations above the mean is shown (green). The compound is shown as sticks (yellow), bound between the surface of the β-TrCP β-propeller (light blue) and the monophosphorylated β-Catenin degron peptide (magenta sticks).

(B) Same as (A) shown for NRX-103094.

## Supplementary Figure 6

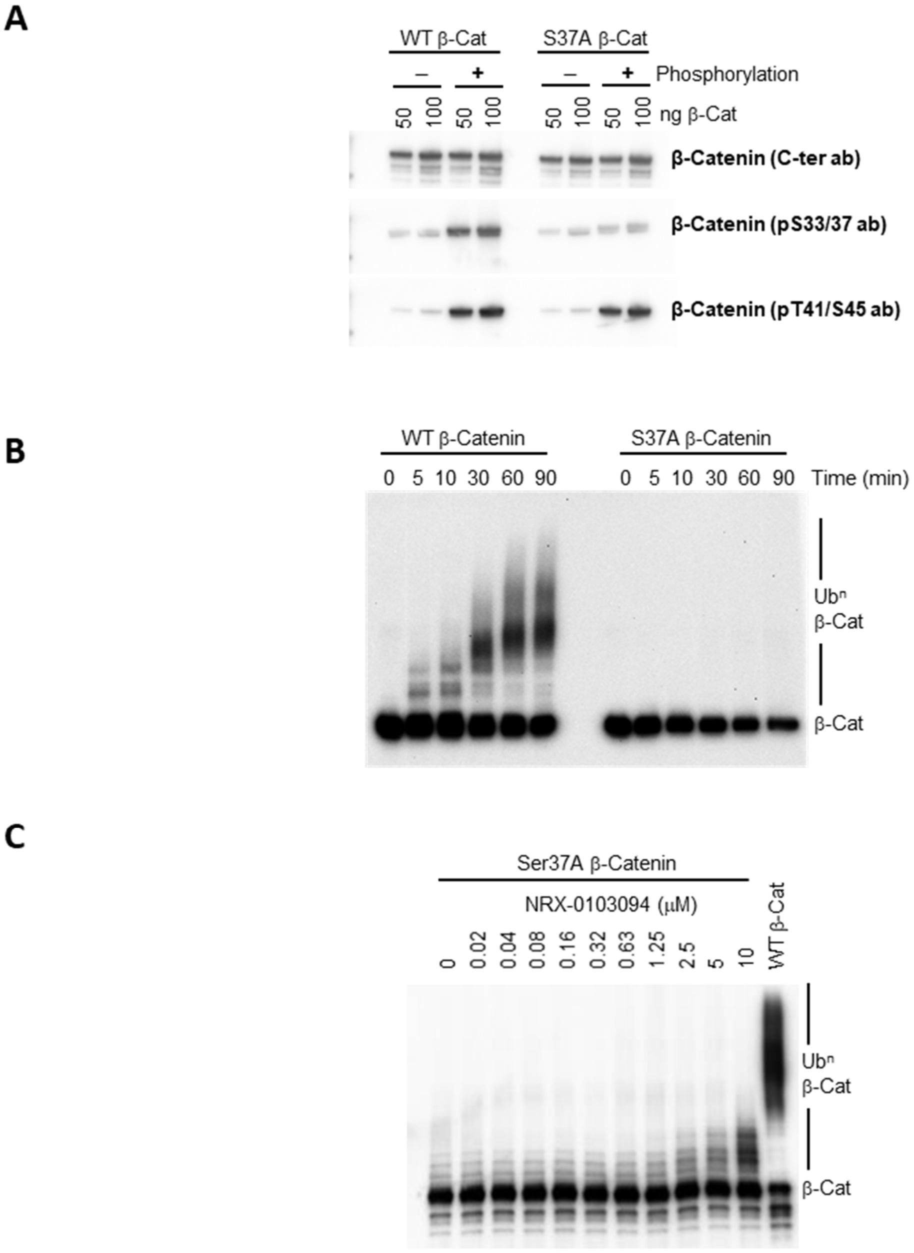


**Supplementary Figure 6: Ubiquitylation of full-length β-Catenin protein by SCF$^{\beta\text{-TrCP}}$.**

(A) 2 μM purified full-length β-Catenin protein, WT or S37A mutant, were phosphorylated with the mix of 2 μM GSK3, 200 nM CK1 and 50 nM Axin. Indicated amounts of the reaction resolved by SDS-PAGE and analyzed by immuno-blotting with specific β-catenin antibodies.

(B) Ubiquitylation of full-length WT or S37A mutant β-Catenin by $SCF^{\beta\text{-TrCP}}$. The reactions were resolved by SDS-PAGE and analyzed by immuno-blotting with β-Catenin C-terminal antibody.

(C) Ubiquitylation of full-length S37A mutant β-Catenin protein by $SCF^{\beta\text{-TrCP}}$ with the increasing concentrations of NRX-103094. Reactions were resolved by SDS-PADE and analyzed by immuno-blotting with β-Catenin C-terminal antibody.

# Supplementary Figure 7

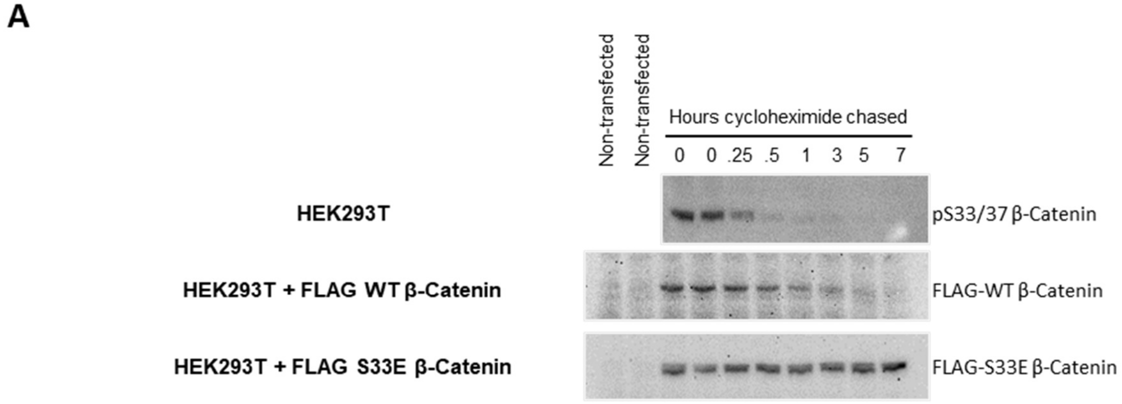


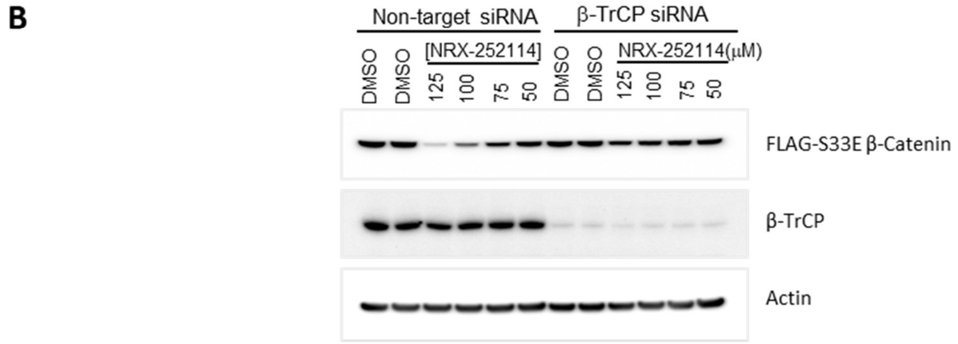


**Supplementary Figure 7: Characterization of engineered HEK293T cell lines stably expressing S33E/S37A β-Catenin.**

(A) HEK293T cells either WT or stably expressing tagged β-Catenin protein (as indicated) were monitored following 100 μg/ml cycloheximide treatment. Cells were harvested at time points indicated and analyzed for protein levels be western analysis.

(B) HEK293T cells stably expressing Myc/FLAG-tagged S33E/S37A β-Catenin were transfected with β-TrCP1&2 siRNAs for 72 hours followed by 6 hours of compound treatment. Cells were then harvested and examined for levels of S33E/S37A β-Catenin and β-TrCP levels by western analysis with antibodies as indicated.

## Supplementary Figure 8

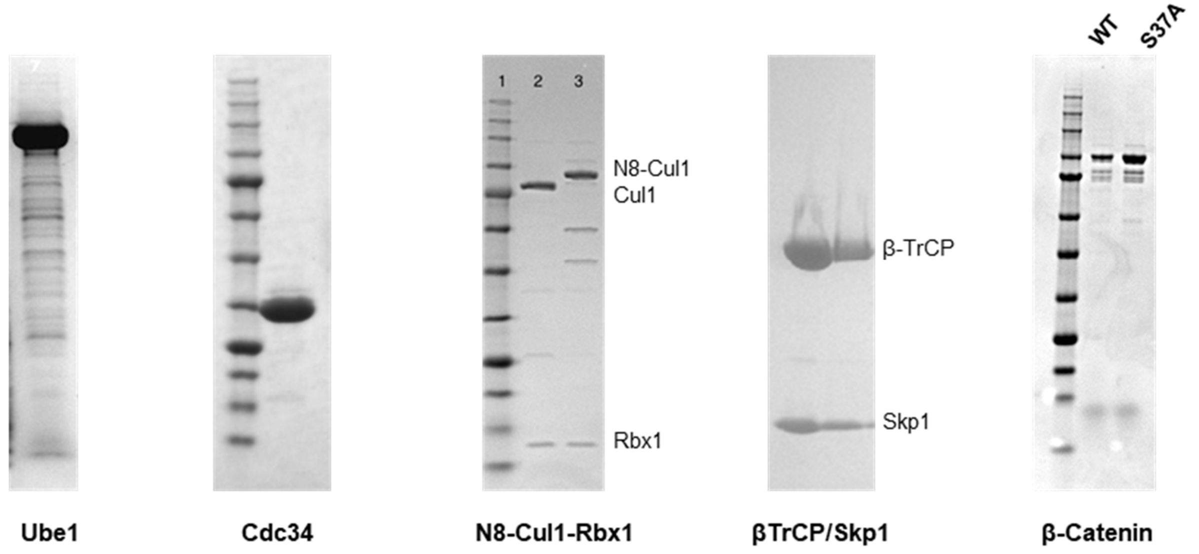


**Supplementary Figure 8: Purified proteins used in this study (Coomassie gels).**

## Supplementary Figure 9

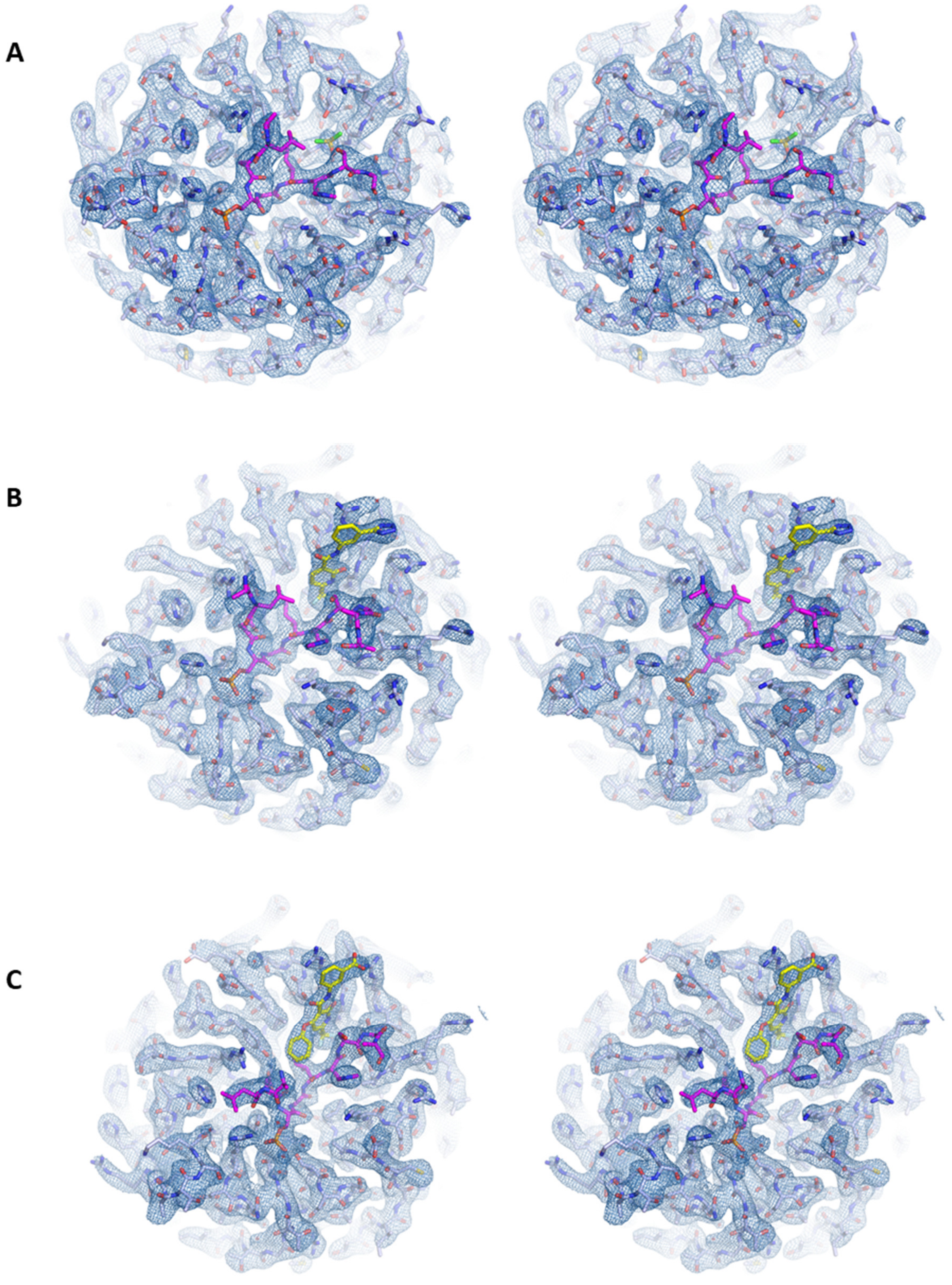

D

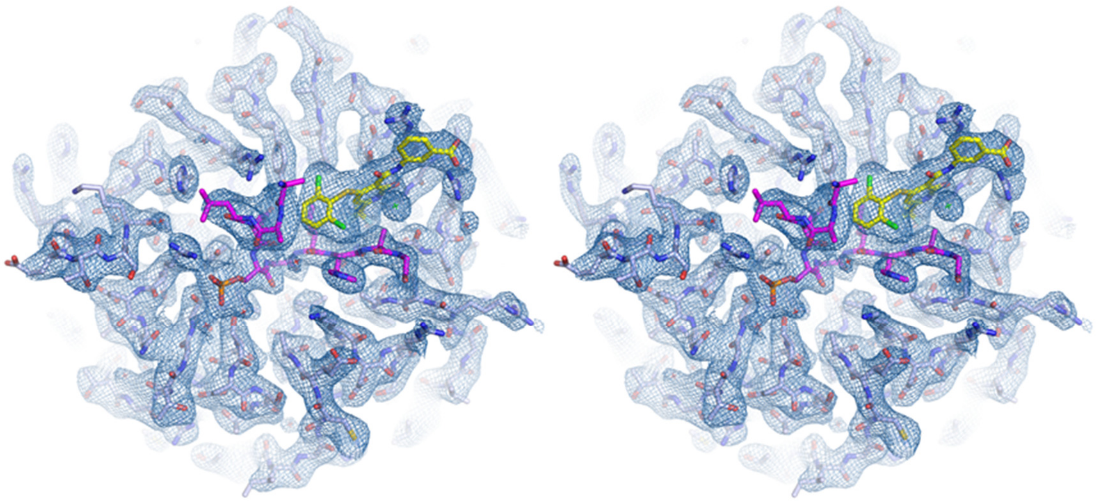

E

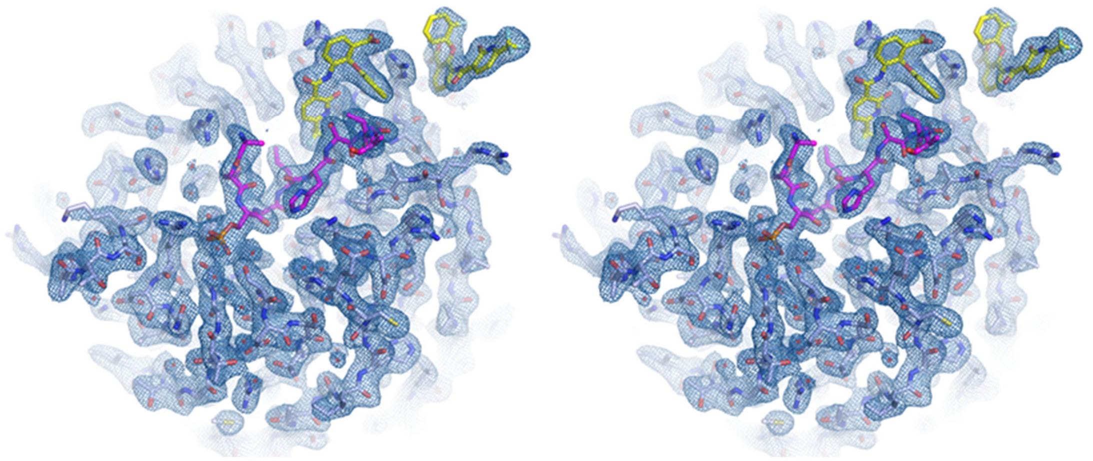

**Supplementary Figure 9: Electron density for the β-TrCP:β-Catenin:Ligand interactions.**

Stereo views of the 2Fo-Fc electron density map (blue mesh), contoured at 1.0 standard deviations above the mean, are shown for the β-TrCP β-propeller (light blue sticks), β-Catenin peptide (magenta sticks), and the compound (yellow sticks) from the (A) pSer33/Ser37 only, (B) NRX-1933, (C) NRX-2663, (D) NRX-103094, and (E) NRX-2776 structures.

# Supplementary Table 1

Data collection and refinement statistics

| | NRX-2776 | NRX-2663 | NRX-103094 | NRX-1933 | pSer33/Ser37 |
|---|---|---|---|---|---|
| **Data collection** | | | | | |
| Space group | $P3_1$ | $P3_1$ | $P3_1$ | $P3_1$ | $P3_1$ |
| Cell dimensions | | | | | |
| *a*, *b*, *c* (Å) | 82.8, 82.8, 111.8 | 82.6, 82.6, 111.1 | 82.5, 82.5, 112.0 | 82.5, 82.5, 111.3 | 82.4, 82.4, 112.9 |
| α, β, γ (°) | 90, 90, 120 | 90, 90, 120 | 90, 90, 120 | 90, 90, 120 | 90, 90, 120 |
| Resolution (Å) | 50.00-2.05 (2.10-2.05) | 50.00-2.35 (2.41-2.35) | 50.00-2.40 (2.46-2.40) | 50.00-2.50 (2.57-2.50) | 50.00-2.70 (2.77-2.70) |
| CC½ | 99.9 (33.3) | 99.9 (38.2) | 99.9 (39.7) | 99.1 (31.9) | 99.7 (34.5) |
| $R_{merge}$ | 4.3 (143.4) | 4.6 (152.6) | 4.4 (139.1) | 11.5 (194.0) | 6.5 (188.5) |
| $I / \sigma I$ | 18.33 (1.09) | 18.29 (0.92) | 17.69 (0.99) | 10.91 (0.94) | 12.38 (0.83) |
| Completeness (%) | 99.2 (98.8) | 99.9 (100.0) | 99.5 (99.4) | 99.5 (100.0) | 99.1 (99.7) |
| Redundancy | 4.8 (4.8) | 4.8 (4.5) | 4.2 (4.2) | 4.1 (4.4) | 4.5 (4.1) |
| **Refinement** | | | | | |
| Resolution (Å) | 27.69-2.05 | 41.29-2.35 | 41.24-2.40 | 41.26-2.50 | 44.26-2.70 |
| No. reflections | 53263 | 35243 | 33154 | 29168 | 23440 |
| $R_{work}$ / $R_{free}$ | 17.85/21.18 | 18.89/22.07 | 19.14/21.44 | 21.35/22.99 | 20.30/23.72 |
| No. atoms | 4413 | 4296 | 4280 | 4167 | 4163 |
| Protein | 4272 | 4224 | 4217 | 4123 | 4154 |
| Ligand/ion | 67 | 35 | 38 | 30 | 9 |
| Water | 74 | 37 | 25 | 14 | -- |
| B-factors | 66.87 | 85.72 | 84.33 | 90.14 | 118.06 |
| Protein | 67.13 | 85.98 | 84.49 | 90.31 | 118.02 |
| Ligand/ion | 62.49 | 73.33 | 78.33 | 75.72 | 135.12 |
| Water | 55.77 | 68.17 | 67.87 | 69.52 | -- |
| R.m.s. deviations | | | | | |
| Bond lengths (Å) | 0.005 | 0.007 | 0.004 | 0.007 | 0.003 |
| Bond angles (°) | 1.02 | 1.26 | 1.08 | 1.27 | 0.92 |

*highest resolution shell shown in parentheses

# Supplementary Table 2

**A**

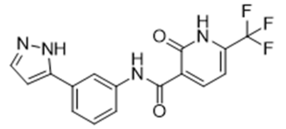

NRX-0001532
MW = 348
# heavy atoms = 25
PSA = 83
cLogP = 1.7

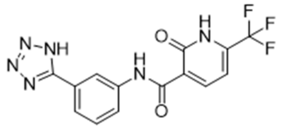

NRX-0001933
MW = 350
# heavy atoms = 25
PSA = 107
cLogP = 0.98

**B**

**$EC_{50}$ for β-TrCP binding**

| Mutant | NRX-0002663 | NRX-0103095 |
|---|---|---|
| pSer33/Ser37 | 80.4 ± 4.4 μM | 0.641 ± .033 μM |
| pSer33/S37A | 22.9 ± 1.4 μM | 0.163 ± 0.006 μM |
| pSer33/S37C | 43.4 ± 7.1 μM | 2.38 ± 0.08 μM |
| pSer33/S37F | No enhancement | 7.4 ± 1.5 μM |

**C**

| Compound | $K_{sol}$ (μM) | Caco-2 (AB/BA) (*$10^{-6}$ cm/sec) | Protein Binding (% Bound) | LogD | PSA |
|---|---|---|---|---|---|
| NRX-252114 | 422 | 3.1/6.7 | 98 | 2.7 | 73 |
| NRX-252262 | 263 | 1.0/6.9 | 99 | 2.7 | 68 |

# Compound Synthesis

**General Chemistry Methods**. $^{1}$H NMR spectra were obtained on Bruker Ascend™ 500 MHz or Bruker AVANCE™ 400 MHz or 300 MHz spectrometers. Chemical shifts are reported in parts per million (ppm). The abbreviations s = singlet, d = doublet, t = triplet, q = quartet, m = multiplet and br = broad are used throughout. Electrospray mass spectra were determined using a Waters Acquity I-Class UPLC electrospray system, a SHIMADZU LCMS-2020 system, an Agilent 1200\G6110A system, or an Agilent 1100\G1956A system. Reaction progress was monitored by thin layer chromatography (TLC) using Analtech 0.25 mm pre-coated glass plates, visualized with UV light, $I_2$, or $KMnO_4$ stain. Flash chromatography was performed using a Teledyne ISCO CombiFlash® RF+ Lumen system with ELSD detection or Combiflash® RF150, using preloaded RediSep® Rf silica columns. Preparative HPLS was performed using an automated UV/Vis and mass-triggered system consisting of the following Waters Modules: 2545 Gradient Module, 515 HPLC Pump, 2489 UV/Vis Detector, Acquity QDa Detector, and 2767 Sample Manager, or a Gilson GX-281 system, a Gilson GX-215 system, or a Gilson-Shimadzu (MS-Triggered) system. Microwave heating was performed using a Biotage® Initiator⁺ that was used in the standard configuration as delivered, and all microwave experiments were carried out in sealed microwave process vials. Solvents and reagents were purchased from commercial vendors and were used as supplied, unless otherwise stated.

**Synthetic Procedures**

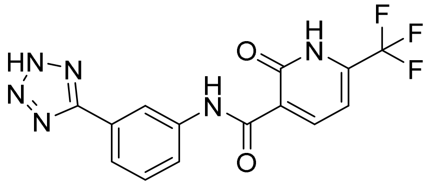


**Preparation of NRX-1933 (2):** A solution of 2-oxo-6-(trifluoromethyl)-1,2-dihydropyridine-3-carboxylic acid (100 mg, 0.48 mmol), HATU (238 mg, 0.62 mmol), diisopropylethylamine (124 mg, 0.96 mmol) and 3-(2H-tetrazol-5-yl)aniline (158 mg, 0.43 mmol) in DMF (1.5 mL) was stirred at room temperature for 4 h. The reaction mixture was diluted with 1.0 N aqueous HCl and extracted with ethyl acetate. The combined organic layer was washed with water and brine, dried over $Na_2SO_4$, and concentrated under vacuum The residue was purified by flash chromatography with 10–25% ethyl acetate/ petroleum ether to give the title compound (36 mg, 0.10 mmol, 23% yield) as light yellow solid.$^{1}$H NMR (500 MHz, DMSO-$d_6$) δ 11.91 (s, 2H), 8.46 (t, J = 1.9 Hz, 1H), 8.39 (d, J = 7.5 Hz, 1H), 7.91 (dd, J = 7.9, 2.1 Hz, 1H), 7.79 (dd, J = 7.9, 1.4 Hz, 1H), 7.60 (t, J = 7.9 Hz, 1H), 7.22 (s, 1H); $^{13}$C NMR (126 MHz, DMSO) δ 163.61, 162.99,

156.09, 142.49, 139.80, 133.79, 130.58, 125.64, 122.74, 122.67, 119.94, 118.48, 114.52, 108.06; $^{19}F$ NMR (471 MHz, DMSO) δ -66.62; LCMS (ESI): m/z: 351.1 [M+1].

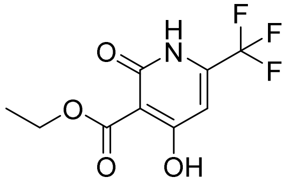

**Preparation of ethyl 4-hydroxy-2-oxo-6-(trifluoromethyl)-1,2-dihydropyridine-3-carboxylate:** To a solution of ethyl (Z)-3-amino-4,4,4-trifluorobut-2-enoate (100 g, 546.1 mmol) and pyridine (65 g, 821 mmol) in $CH_2Cl_2$ (1 L) was added ethyl 3-chloro-3-oxopropanoate (115 g, 763 mmol) dropwise at 0 °C over 1 h. After stirring at room temperature for 16 h, the reaction mixture was diluted with water (1 L), and extracted with $CH_2Cl_2$. The combined organic layer was washed with 1N aqueous HCl and saturated aqueous $NaHCO_3$, dried over $Na_2SO_4$, and concentrated under vacuum to afford ethyl (Z)-3-(3-ethoxy-3-oxopropanamido)-4,4,4-trifluorobut-2-enoate (200 g) as a brown oil, which was used in the next step without further purification.

To a solution of ethyl (*Z*)-3-(3-ethoxy-3-oxopropanamido)-4,4,4-trifluorobut-2-enoate (200 g, 672.9 mmol) in ethanol (1 L) was added *t*-BuONa (65 g, 676 mmol) in portions under $N_2$. After stirring at 60 $^oC$ for 18 h, the reaction mixture was concentrated under vacuum, and the residue was suspended in saturated aqueous citric acid. After stirring for 1 h, the suspension was filtered, washed with saturated aqueous citric acid and water, and dried to afford ethyl 4-hydroxy-2-oxo-6-(trifluoromethyl)-1,2-dihydropyridine-3-carboxylate (80 g, 58%) as a light yellow solid. $^1H$ NMR (300 MHz, DMSO-$d_6$) δ 12.00 (br, 2H), 6.78 (s, 1H), 4.24 (q, $J$ = 7.2 Hz, 2H), 1.25 (t, $J$ = 7.2 Hz, 3H); MS (ESI) m/z = 252 [M+1].

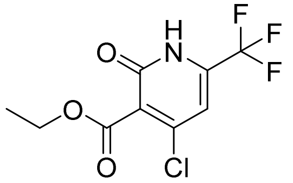

**Preparation of ethyl 4-chloro-2-oxo-6-(trifluoromethyl)-1,2-dihydropyridine-3-carboxylate:** ethyl 4-hydroxy-2-oxo-6-(trifluoromethyl)-1,2-dihydropyridine-3-carboxylate (240 g, 955.6 mmol) was added to a solution of $PhPOCl_2$ (926.1 g, 4.77 mol) in portions at room temperature. After stirring at 100 $^oC$ for 16 h, the reaction mixture was cooled to room temperature, and poured into ice water. After stirring for 1 h, the resulting mixture was extracted with ethyl acetate. The combined organic layer was washed with

saturated aqueous $NaHCO_3$ and brine, dried over $Na_2SO_4$, and concentrated under vacuum. The residue was purified by flash chromatography with 0–20% ethyl acetate/ petroleum ether to afford the titled compound (65 g, 25%) as a white solid. $^1$H NMR (300 MHz, DMSO-$d_6$) δ 12.24 (s, 1H), 7.64 (s, 1H), 4.37 (q, $J$ = 7.2 Hz, 2H), 1.30 (t, $J$ = 7.2 Hz, 3H); MS (ESI) m/z = 270 [M+1].

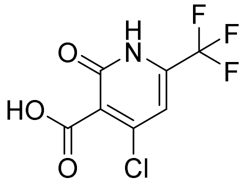

**Preparation of 4-chloro-2-oxo-6-(trifluoromethyl)-1,2-dihydropyridine-3-carboxylic acid:** The mixture of ethyl 4-chloro-2-oxo-6-(trifluoromethyl)-1,2-dihydropyridine-3-carboxylate (1.50 g, 5.56 mmol) in MeOH (20 mL), THF (20 mL) and water (10 mL) was added $LiOH{\cdot}H_2O$ (933 mg, 22.3 mmol). After stirring for18 h, the volatile was removed under vacuum, and the reaction mixture was filtered. The filtrate was acidified with 1N aqueous HCl, and extracted with ethyl acetate. The combined organic layer was dried over $Na_2SO_4$, and concentrated under vacuum to afford the titled compound (1.30 g, 5.38 mmol, 97% yield) as a white solid. $^1$H NMR (300MHz, DMSO-$d_6$) δ 7.09 (s, 1H); MS (ESI) m/z = 242 [M+1].

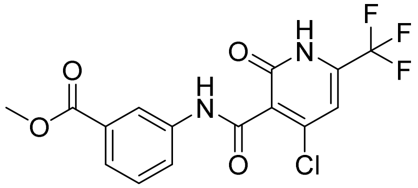

**Preparation of methyl 3-(4-chloro-2-oxo-6-(trifluoromethyl)-1,2-dihydropyridine-3-carboxamido)benzoate:** To the solution of 4-chloro-2-oxo-6-(trifluoromethyl)-1,2-dihydropyridine-3-carboxylic acid (1 g, 4.14 mmol) and HATU (2.05 g, 5.38 mmol) in DMF (10 mL) was added DIEA (1.07 g, 8.28 mmol) and methyl 3-aminobenzoate (563 mg, 3.73 mmol). After stirring at room temperature for 4 h, the reaction mixture was diluted with 1N aqueous HCl, and extracted with ethyl acetate. The combined organic layer was washed with water and brine, dried over $Na_2SO_4$, and concentrated under vacuum. The residue was purified by flash chromatography with 10–30% ethyl acetate/ petroleum ether to afford the titled compound (350 mg, 934 µmol, 23%) as a light yellow solid. $^1$H NMR (400MHz, DMSO-$d_6$) δ 13.07 (br. s., 1H), 10.90 (s, 1H), 8.39 (t, $J$=1.6 Hz, 1H), 7.90 - 7.80 (m, 1H), 7.77 - 7.65 (m, 2H), 7.57 - 7.49 (m, 1H), 3.88 (s, 3H); MS (ESI) m/z = 375 [M+1].

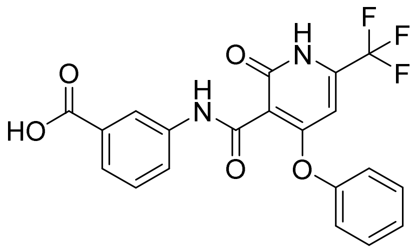

**Preparation of NRX-2663 (3):** To a solution of phenol (50 mg, 533 μmol) and methyl 3-(4-chloro-2-oxo-6-(trifluoromethyl)-1,2-dihydropyridine-3-carboxamido)benzoate (100 mg, 266 μmol) in NMP (3 mL) was added $K_2CO_3$ (73.7 mg, 533 μmol). After stirring at 100 °C for 18 h, the reaction mixture was diluted with ethyl acetate, washed with saturated aqueous $Na_2CO_3$, water and brine, dried over $Na_2SO_4$, and concentrated under vacuum to afford methyl 3-(2-oxo-4-phenoxy-6-(trifluoromethyl)-1,2-dihydropyridine-3-carboxamido)benzoate (184 mg, 426 μmol, 80%) as a yellow oil.
To a solution of methyl 3-(2-oxo-4-phenyl-6-(trifluoromethyl) 1,2-dihydropyridine-3-carboxamido)benzoate (100 mg, 231 μmol) in MeOH (2 mL), THF (2 mL) and water (1 mL) was added $LiOH{\cdot}H_2O$ (77.6 mg, 1.85 mmol). After stirring at room temperature for 18 h, the reaction mixture was acidified with 1N aqueous HCl to pH = 5–6, and extracted with ethyl acetate. The combined organic layer was dried over $Na_2SO_4$, and concentrated under vacuum. The residue was purified by HPLC using acetonitrile/ water with formic acid to afford the titled compound (53.0 mg, 126 μmol, 55%) as a yellow solid. $^1$H NMR (400MHz, methanol-$d_4$) δ 10.80 (s, 1H), 8.34 (s, 1H), 7.86 (d, $J$ = 8.0 Hz, 1H), 7.68 (d, $J$ = 7.5 Hz, 1H), 7.58–7.43 (m, 3H), 7.39–7.29 (m, 1H), 7.25 (d, $J$ = 8.0 Hz, 2H), 6.58 (s, 1H); $^{13}$C NMR (126 MHz, DMSO) δ 167.50, 163.99, 163.19, 161.30, 154.06, 139.53, 131.91, 131.07, 129.66, 126.52, 125.06, 123.69, 122.31, 121.01, 120.29, 120.12, 103.35, 101.61;$^{19}$F NMR (471 MHz, DMSO) δ -67.30; MS (ESI) m/z = 419 [M+1].

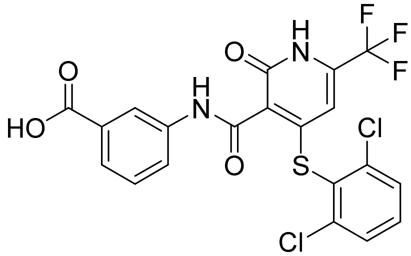

**Preparation of NRX-103094 (4):** To a mixture of methyl 3-[[4-chloro-2-oxo-6-(trifluoromethyl)-1H-pyridine-3-carbonyl]amino]benzoate (150 mg, 400 μmol) and 2,6-dichlorobenzenethiol (107 mg, 600 μmol) in dioxane (8 mL) was added $K_2CO_3$ (110 mg, 800 μmol). The mixture was stirred at 110 °C for 24 h. The mixture was cooled to room temperature, and concentrated under vacuum. The residue was

stirred in saturated aqueous $NH_4Cl$ solution for 1 min. The aqueous phase was extracted with ethyl acetate twice. The combined organic layer was washed with brine, dried over $Na_2SO_4$, and concentrated under vacuum. The residue was loaded on a silica gel plug, and eluted with 10% methanol in dichloromethane to afford methyl 3-[[4-(2,6-dichlorophenyl)sulfanyl-2-oxo-6-(trifluoromethyl)-1H-pyridine-3-carbonyl]amino] benzoate (130 mg, 251 mmol, 63% yield) as a white solid.

To a mixture of methyl 3-[[4-(2,6-dichlorophenyl)sulfanyl-2-oxo-6-(trifluoromethyl)-1H-pyridine-3-carbonyl]amino]benzoate (120 mg, 232 µmol) in MeOH (5 mL) and $H_2O$ (1 mL) was added NaOH (28 mg, 696 µmol). The mixture was stirred at 50°C for 2 h. The mixture was cooled to room temperature, concentrated under vacuum, and acidified to pH 3 with aq 1 N HCl. The aqueous phase was extracted with ethyl acetate three times. The combined organic layer was washed with brine, dried with $Na_2SO_4$, and concentrated in vacuum. The residue was purified by HPLC using acetonitrile/ water with TFA to afford the titled compound (47 mg, 0.94 mmol, 40% yield) as a white solid. $^1$H NMR (400 MHz, DMSO-*d*$_6$) δ 11.33 (s, 1H), 8.39 (s, 1H), 7.89 (d, *J* = 8.1 Hz, 1H), 7.74 (dd, *J* = 19.8, 7.9 Hz, 4H), 7.63 (t, *J* = 8.1 Hz, 1H), 7.51 (t, *J* = 7.9 Hz, 1H), 6.17 (s, 1H); $^{13}$C NMR (126 MHz, $CDCl_3$) δ 168.94, 167.07, 166.36, 162.72, 142.62, 140.82, 138.89, 132.17, 131.44, 130.68, 129.71, 129.67, 123.87, 123.85, 123.61, 120.24, 120.10, 105.12; $^{19}$F NMR (471 MHz, DMSO) δ -67.52; MS (ESI) m/z = 503 [M+1].

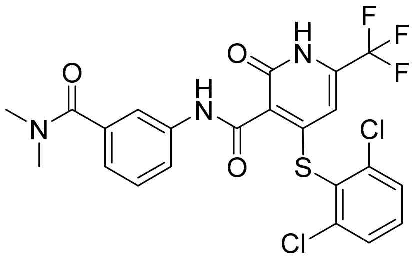


**Preparation of NRX-103095 (5):** To a solution of 4-chloro-2-oxo-6-(trifluoromethyl)-1,2-dihydropyridine-3-carboxylic acid (3 g, 12.4 mmol), HOAt (2.03 g, 14.9 mmol) and EDCI (2.86 g, 14.9 mmol) in $CH_2Cl_2$ (20 mL) was added *N*-methylmorpholine (6.83 mL, 62.1 mmol) and 3-amino-*N*,*N*-dimethyl-benzamide (2.04 g, 12.4 mmol). After stirring at room temperature for 4 h, the reaction mixture was washed with 1N aqueous HCl, dried over $Na_2SO_4$, and concentrated in vacuo to afford 4-chloro-*N*-(3-(dimethylcarbamoyl)phenyl)-2-oxo-6-(trifluoromethyl)-1,2-dihydropyridine-3-carboxamide (2.72 g, 6.1 mmol, 49%) as a pale white solid.

To a solution of 4-chloro-*N*-(3-(dimethylcarbamoyl)phenyl)-2-oxo-6-(trifluoromethyl)-1,2-dihydro pyridine-3-carboxamide (350 mg, 903 µmol) and 2,6-dichlorobenzenethiol (194 mg, 1.08 mmol) in dioxane (16 mL) was added $K_2CO_3$ (250 mg, 1.81 mmol) under $N_2$. After stirring at 100°C for 18 h, the

reaction mixture was cooled to room temperature, and poured into saturated aqueous $NH_4Cl$. The aqueous phase was extracted with ethyl acetate. The combined organic layer was washed with brine, dried over $Na_2SO_4$, and concentrated under vacuum. The residue was purified by HPLC using acetonitrile/ water with TFA to afford the titled compound (217 mg, 405 μmol, 45%) as a white solid. $^1$H NMR (400 MHz, Methanol-$d_4$) δ 7.91 (t, $J$ = 1.8 Hz, 1H), 7.79 – 7.65 (m, 3H), 7.58 (dd, $J$ = 8.9, 7.3 Hz, 1H), 7.49 (t, $J$ = 7.9 Hz, 1H), 7.23 (dt, $J$ = 7.6, 1.3 Hz, 1H), 6.03 (s, 1H), 3.15 (s, 3H), 3.08 (s, 3H); $^{13}$C NMR (126 MHz, Methanol-$d_4$) δ 169.71, 162.06, 161.28, 140.76, 138.46, 137.23, 137.20, 133.60, 129.81, 129.76, 128.91, 127.37, 122.34, 120.23, 119.43, 117.98, 117.93, 106.68, 34.70; $^{19}$F NMR (471 MHz, Methanol-$d_4$) δ -67.35; MS (ESI) m/z = 530 [M+1].

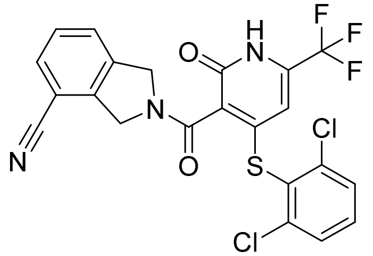


**Preparation of NRX-252114 (6):** A mixture of 4-[(2,6-dichlorophenyl)sulfanyl]-2-oxo-6-(trifluoromethyl)-1,2-dihydropyridine-3-carboxylic acid (19 mg, 0.05 mmol) and $SOCl_2$ (0.5 mL) was stirred at 70 °C for 2 h. The reaction mixture was cooled to room temperature, and concentrated under vacuum. The residue was dissolved in $CH_2Cl_2$ (0.5 mL), and added dropwise to a solution of isoindoline-4-carbonitrile hydrochloride (30 mg, 0.17 mmol) and DIEA (35 μL, 0.17 mmol) in $CH_2Cl_2$ (0.5 mL) at 0 °C. After stirring at room temperature for 16 h, the reaction mixture was concentrated under vacuum. The residue was purified by HPLC using acetonitrile/ water with formic acid to afford the titled compound (14 mg, 56%) as a white solid. $^1$H NMR (500 MHz, DMSO-$d_6$) δ 12.89 (s, 1H), 7.88 – 7.60 (m, 4H), 7.60 – 7.40 (m, 2H), 6.28 (s, 1H), 5.02 – 4.54 (m, 4H); $^{13}$C NMR (126 MHz, DMSO) δ 163.09, 163.05, 160.92, 148.99, 141.17, 141.10, 139.90, 138.40, 138.11, 134.11, 132.02, 130.33, 130.27, 129.28, 128.99, 126.52, 117.16, 107.02, 52.73, 51.56; $^{19}$F NMR (471 MHz, DMSO) δ -67.37; MS m/z = 510 [M+1].

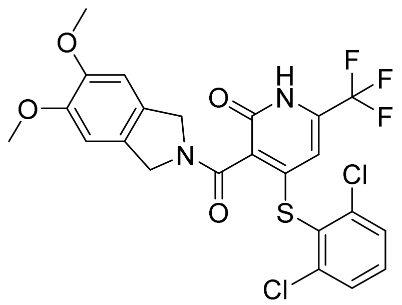


**Preparation of NRX-252262 (7):** A solution of 4-((2,6-dichlorophenyl)thio)-2-oxo-6-(trifluoromethyl)-1,2-dihydropyridine-3-carboxylic acid (2.0 g, 5.21 mmol) in $SOCl_2$ (50 mL) was stirred at 70 °C for 2 h before concentrated under vacuum. The residue was dissolved in $CH_2Cl_2$ (20 mL), and added dropwise to a solution of 5,6-dimethoxy-2,3-dihydro-1H-isoindole hydrochloride (1.0 g, 4.64 mmol) and DIEA (2.7 mL, 15.48 mmol) in $CH_2Cl_2$ (50 mL) at 0 °C. After stirring at room temperature for 16 h, the reaction mixture was concentrated under vacuum. The residue was purified by reverse phase flash chromatography using acetonitrile/ water with formic acid to afford the titled compound (1.04 g, 37%) as a light brown solid. $^{1}H$ NMR (400 MHz, DMSO-$d_6$) δ 12.89 (s, 1H), 7.77 (d, $J$ = 8.2 Hz, 2H), 7.63 (t, $J$ = 8.2, 1H), 7.03 (s, 1H), 6.99 (s, 1H), 6.32 (s, 1H), 4.78 – 4.77 (m, 2H), 4.68 – 4.41 (m, 2H), 3.77 (s, 3H), 3.71 (s, 3H); $^{13}C$ NMR (126 MHz, DMSO) δ 162.89, 160.54, 149.48, 149.35, 148.85, 141.15, 134.16, 130.30, 127.83, 127.53, 126.49, 107.07, 106.96, 56.15, 56.09, 52.56, 52.12; $^{19}F$ NMR (471 MHz, DMSO) δ -67.37; MS m/z = 545 [M+1].
